# Supplementary material for: Mitochondrial complex III deficiency drives c-MYC overexpression and illicit cell cycle entry leading to senescence and segmental progeria
Source: Nat Commun. 2023 Apr 24;14:2356. doi: 10.1038/s41467-023-38027-1 (PMC10126100; doi:10.1038/s41467-023-38027-1)
Supplement: Supplementary file 1 — Supplementary Information [file 41467_2023_38027_MOESM1_ESM.pdf]

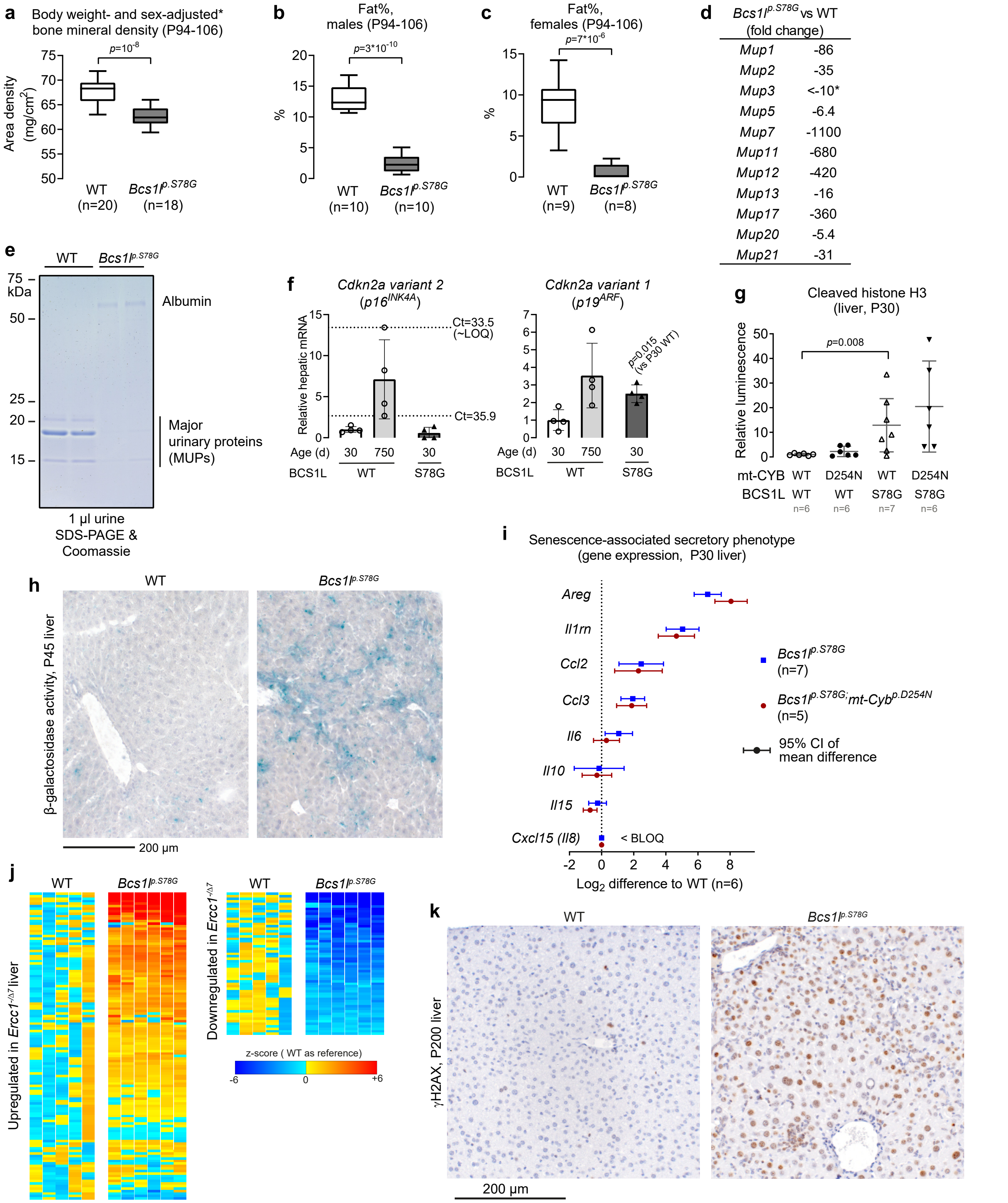


**Supplementary Fig. 1 | Characterization of progeroid phenotypes in CIII-deficient mice. a** Bone mineral density (DEXA) in adult WT and *Bcs1l*$^{p.S78G}$ mice. The data were normalized for sex and body weight using a linear regression model. Skull was excluded from the analyses. The unnormalized data have been previously published[26]. **b, c** Body fat percentage (DEXA) of adult WT and *Bcs1l*$^{p.S78G}$ mice. **d** Hepatic expression (RNASeq) of MUP genes at P150 (n=3 males/genotype). * Below limit of quantification (BLOQ) or not detected in *Bcs1l*$^{p.S78G}$ livers (read counts < 10). **e** Representative SDS-PAGE analysis of albuminuria and MUPs in the urine of adult (P200) WT (n=3) and *Bcs1l*$^{p.S78G}$ (n=4) male mice. **f** qPCR analysis of *Cdkn2a* splice variants (n=4/genotype). P750 WT group was included as a positive control. ~LOQ, approximate limit of quantification. The bar graphs present mean and ±SD. **g** Western blot quantification of cleaved histone H3 in P30 liver lysates. The error bars represent 95%-CI of mean. Representative blot is shown in Fig. 1m. **h** β-galactosidase activity staining of P45 liver cryosections (representative results of 7 mice/genotype). **i** qPCR analysis of genes related to senescence-associated secretory phenotype (SASP). BLOQ, threshold cycles >36. **j** Hepatic gene expression in P150 WT and *Bcs1l*$^{p.S78G}$ mice of genes significantly upregulated or downregulated in the liver of *Ercc1*$^{-/\Delta}$ mice (E-MEXP-1503[40]). **k** P200 liver sections immunostained for γH2AX (representative result of 6 mice/genotype). Statistics: **a-c**, Welch's two-sided t-test; **f, g, i,** 1-way ANOVA followed by the selected pairwise comparisons. The box blots show the median, the quartiles, and the minimum and maximum. All data points derive from independent mice. Source data are provided as a Source Data file.

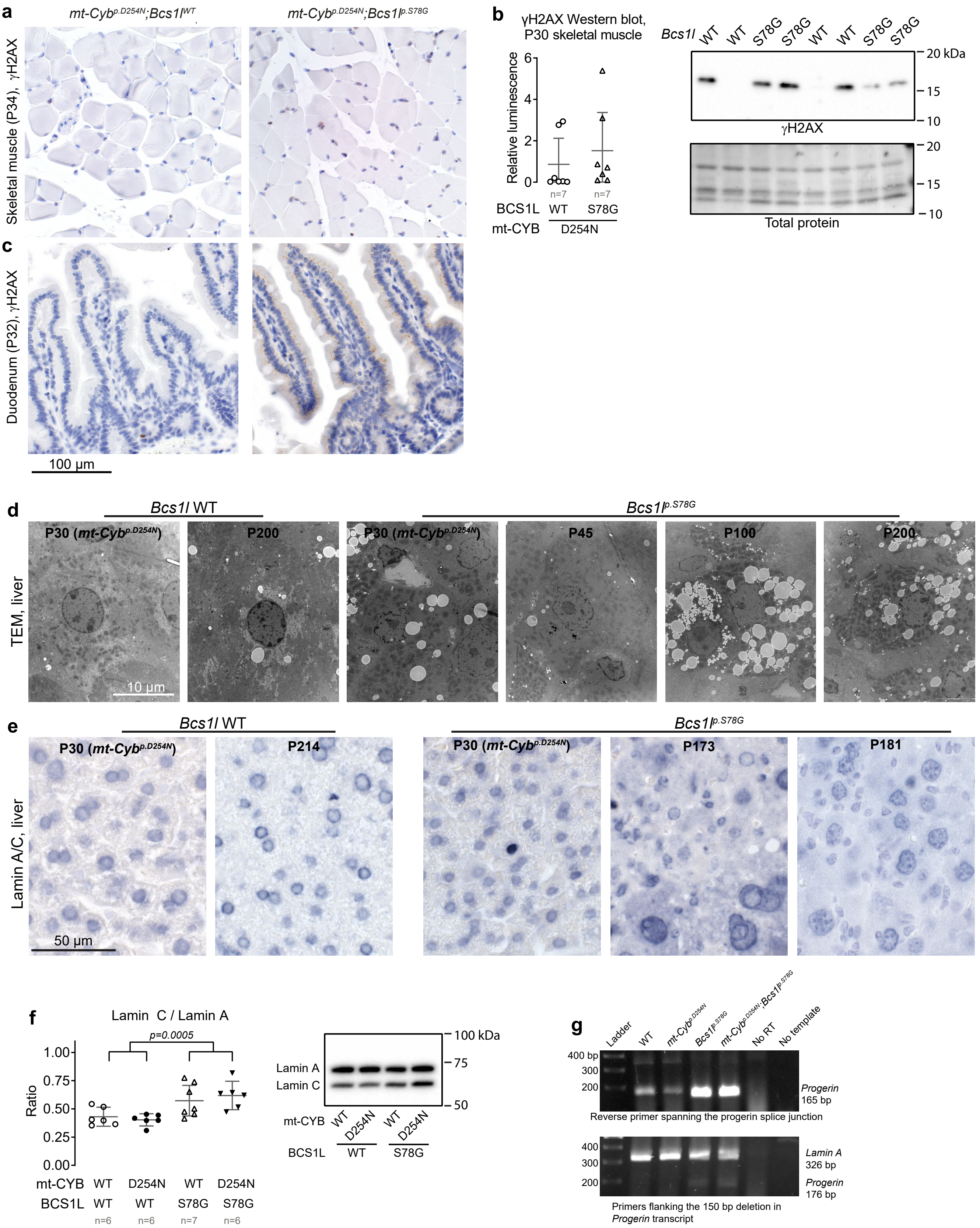


**Supplementary Fig. 2 | Effect of *Bcs1l*$^{p.S78G}$ mutation on DNA damage in skeletal muscle and small intestine, and on nuclear morphology and *Lmna* splicing. a** Skeletal muscle (quadriceps) sections immunostained for γH2AX showing lack of genotype difference in DNA damage in this tissue (representative result of 4 mice/genotype). **b** Western blot quantification of γH2AX in skeletal muscle (whole calf muscle lysate) and a representative blot. **c** γH2AX immunostaining of intestinal villi from duodenum showing lack of nuclear staining in *Bcs1l*$^{p.S78G}$;*mt-Cyb*$^{p.S254N}$ mice (representative result from 9 mutant mice and 5 controls). The antibody showed cytoplasmic granular staining pattern of unknown significance in the enterocytes of *Bcs1l*$^{p.S78G}$;*mt-Cyb*$^{p.S254N}$ mice. **d** Transmission electron microscopy (TEM) images of hepatocytes, the nuclei of which are shown in Fig. 3c at higher magnification. **e** Lamin A/C immunostaining of liver sections (representative micrographs from 13 WTs and 14 mutants). **f** Western blot quantification of lamin C-to-lamin A ratio in P30 liver lysates and a representative blot. **g** Agarose gel electrophoresis of *progerin* and *lamin A* qPCR products (40 cycles). Statistics: Welch's two-sided t-test or 1-way ANOVA followed by the selected pairwise comparisons (Welch's t-statistics). The error bars represent 95% CI of mean. All data points derive from independent mice. Source data are provided as a Source Data file.

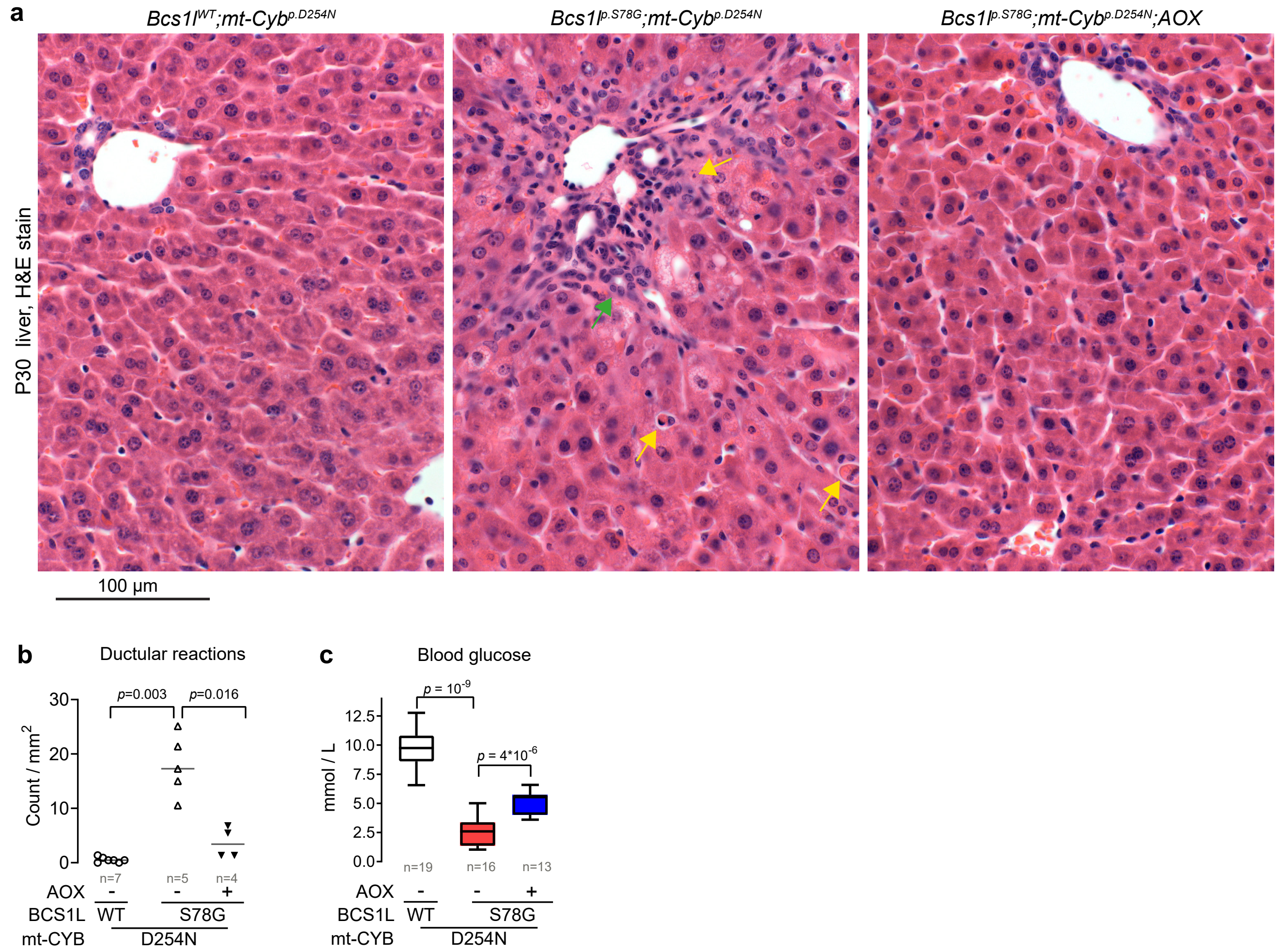


**Supplementary Fig. 3 | AOX expression prevents liver disease in juvenile *Bcs1l*$^{p.S78G}$;*mt-Cyb*$^{p.S254N}$ mice. a** Representative H&E-stained P30 liver sections. Green arrow, increased number of bile duct-like structures (ductular reactions), and non-parenchymal cells surrounding a hepatic portal triad. Yellow arrows, apoptotic cell remnants. **b** Quantification of ductular reactions as a marker of liver disease severity and activation of hepatic progenitor cell response. Horizontal lines represent the mean value. **c** Blood glucose levels with hypoglycemia as an indicator of failing liver function. All data are from 1-month old mice. Statistics: **b**, Kruskal Wallis test followed by the selected Mann-Whitney U tests. **c**, 1-way ANOVA followed by the selected pairwise comparisons (Welch's t-statistics). The box blot shows the median, the quartiles, and the minimum and maximum. All data points derive from independent mice. Source data are provided as a Source Data file.

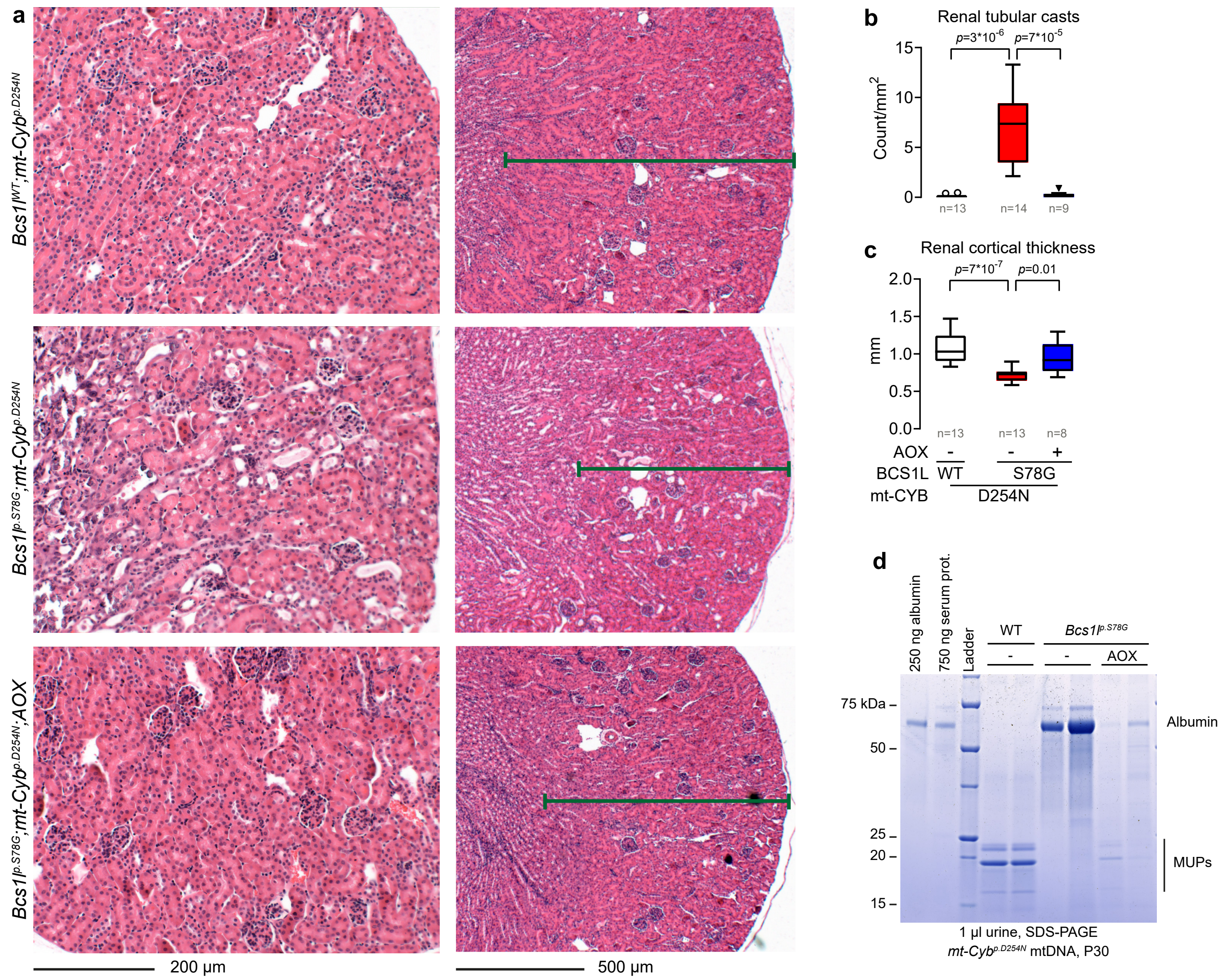


**Supplementary Fig. 4 | AOX expression ameliorates kidney pathology in *Bcs1l^p.S78G^;mt-Cyb^p.S254N^* mice. a** Representative images of H&E-stained renal cortex. **b** Number of renal tubular casts as a measure of renal insufficiency. **c** Measurement of renal cortical thickness. The decreased renal cortical thickness in *Bcs1l$^{p.S78G}$;mt-Cyb$^{p.S254N}$* mice indicates loss of renal tubular mass. **d** Representative SDS-PAGE analysis of urine samples for albuminuria and MUP excretion. All data are from 1-month old mice. Statistics: **b**, Kruskal Wallis test followed by the selected Mann-Whitney U tests. **c**, 1-way ANOVA followed by the selected pairwise comparisons (Welch's t-statistics). The box blots show the median, the quartiles, and the minimum and maximum. All data points derive from independent mice. Source data are provided as a Source Data file.

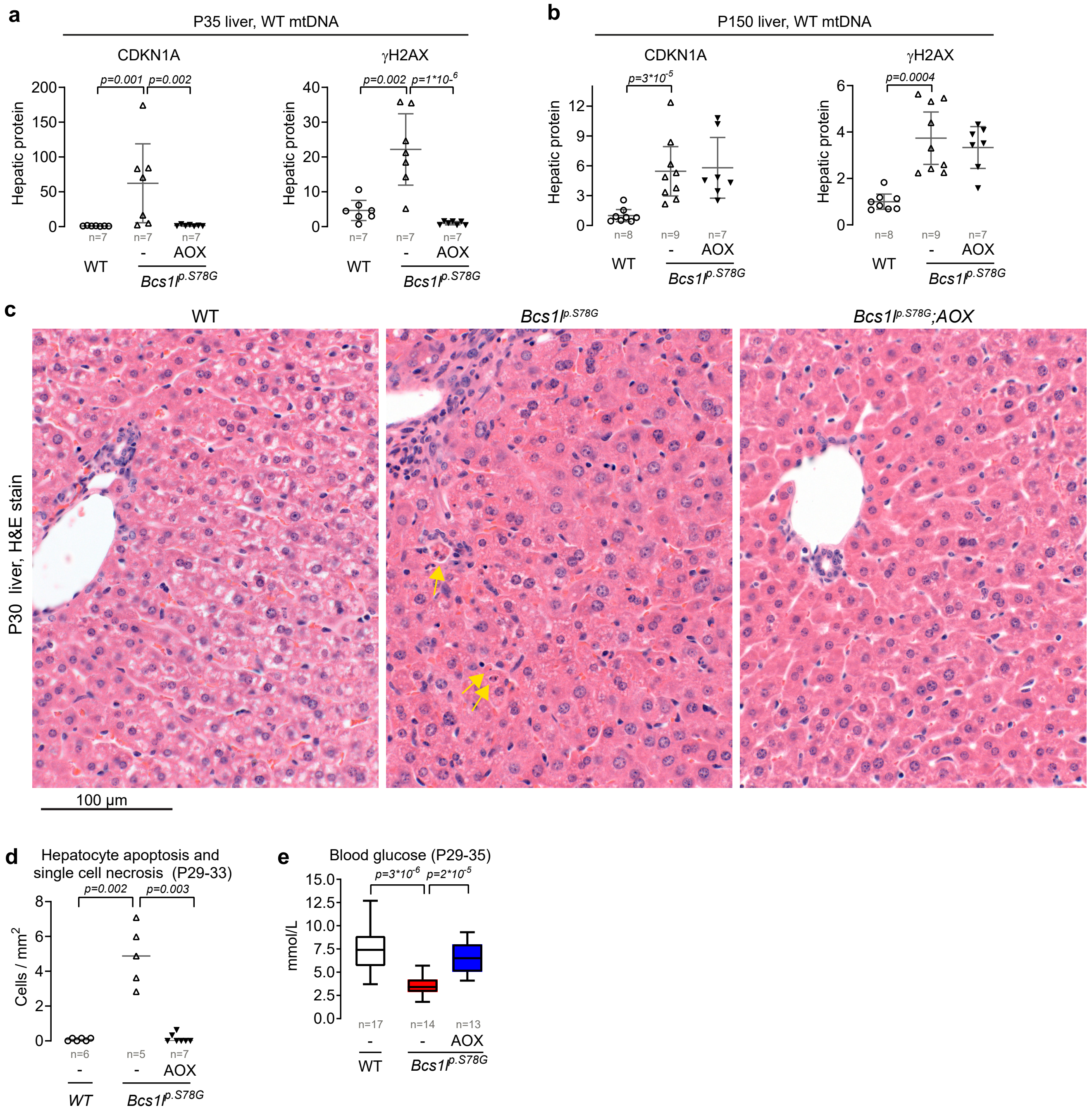


**Supplementary Fig. 5 | AOX expression prevents DNA damage and liver disease in juvenile *Bcs1l*$^{p.S78G}$ mice. a, b** Hepatic levels of CDKN1A and γH2AX in juvenile (**a**) and adult (**b**) WT and *Bcs1l*$^{p.S78G}$ mice. **c** Representative H&E-stained liver sections. The upper left corner of *Bcs1l*$^{p.S78G}$ liver cross section shows a portal triad with increased number of non-parenchymal cells (portal area expansion). Yellow arrows point to apoptotic cell remnants. **d** Quantification of apoptotic and necrotic cells based on cell morphology (H&E stain, Materials and Methods). **e** Blood glucose levels with hypoglycemia as an indicator of failing liver function. Statistics: **a**, **b**, and **e**, 1-way ANOVA followed by the selected pairwise comparisons (Welch's t-statistics). **d**, Kruskal Wallis test followed by the selected Mann-Whitney U tests. The error bars in scatter blots represent 95% CI of mean. The box blot shows the median, the quartiles, and the minimum and maximum. All data points derive from independent mice. Source data are provided as a Source Data file.

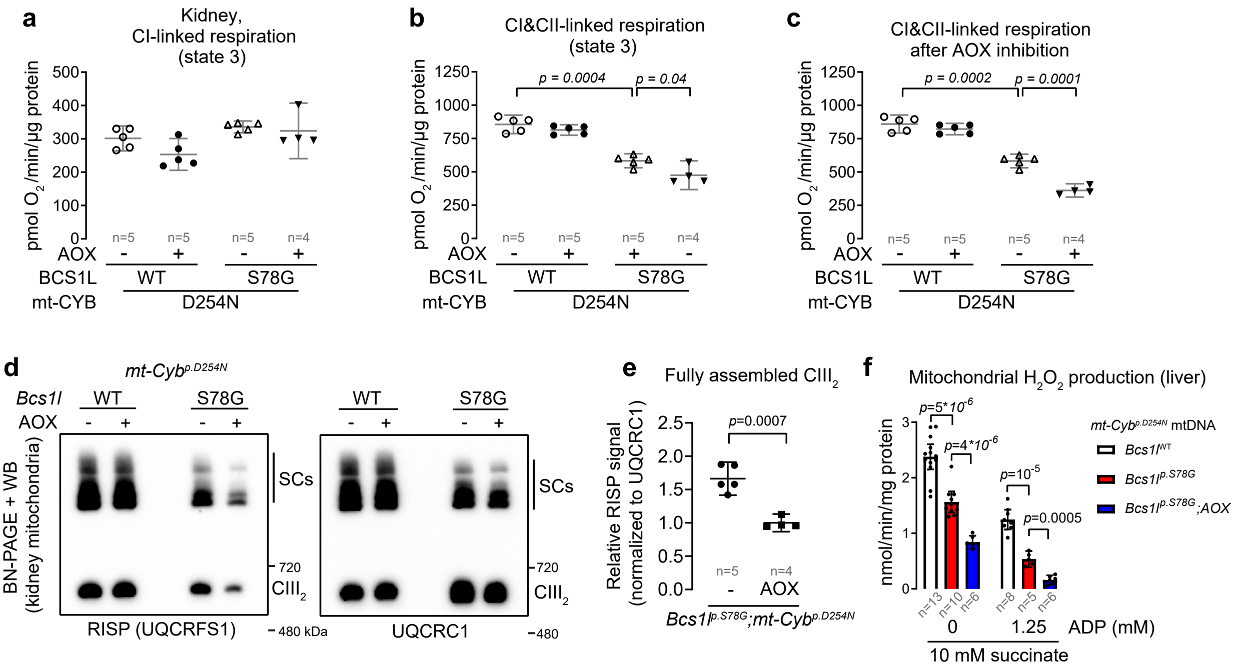


**Supplementary Fig. 6 | AOX exacerbates the CIII assembly defect caused by mutated *Bcs1l*.** **a** Phosphorylating respiration of kidney mitochondria in the presence of NADH-generating substrates (malate, glutamate, and pyruvate) (CI-linked respiration). **b** Phosphorylating respiration of kidney mitochondria in the presence of the NADH-generating substrates and succinate (CI&CII-linked respiration). **c** CI&CII-linked respiration of kidney mitochondria after inhibition of AOX with 50 µM propyl gallate. **d** Representative analysis of CIII assembly using Blue-Native PAGE and Western blot from digitonin-solubilized kidney mitochondria. SCs, supercomplexes containing CIII. **e** Quantification of the relative amount of fully assembled CIII based on the presence of the RISP subunit. **f** Measurement of hepatic mitochondrial $H_2O_2$ production under conditions favouring reverse-electron flow. All data are from 1-month old mice. Statistics: **a**-**c** and **f** 1-way ANOVA followed the selected pairwise comparisons (Welch's t-statistics). **e** Welch's two-sided t-test. The error bars represent 95% CI of mean. All data points derive from independent mice. Source data are provided as a Source Data file.

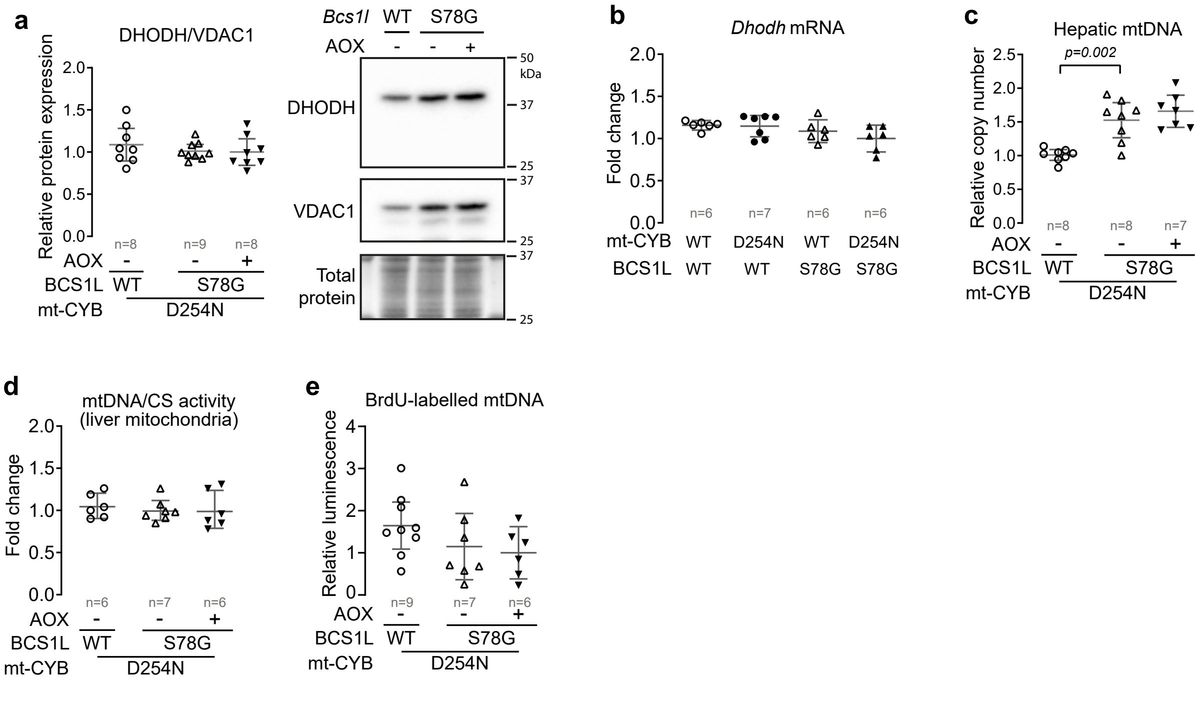


**Supplementary Fig. 7 | Supporting data related Figure 6.** **a** DHODH expression normalized to VDAC1, a reference mitochondrial protein. Hepatic VDAC1 levels are similar to those of several other mitochondrial proteins in *Bcs1l^p.S78G^;mt-Cyb^p.S254N^* mice[17]. **b** Hepatic mRNA expression of *Dhodh*. **c** qPCR analysis of mtDNA copy number from liver DNA. **d** qPCR analysis of relative mtDNA amount in isolated liver mitochondria. The data were normalized to the activity of mitochondrial enzyme citrate synthase (CS). **e** Assessment of hepatic mtDNA replication based on BrdU incorporation into mtDNA during 16h *in vivo* exposure. BrdU was detected from isolated mtDNA using denaturing DNA dot blot and a BrdU-specific antibody. All data are from 1-month old mice. Statistics: 1-way ANOVA followed by the selected pairwise comparisons (Welch's t-statistics). The error bars represent 95% CI of mean. All data points derive from independent mice. Source data are provided as a Source Data file.

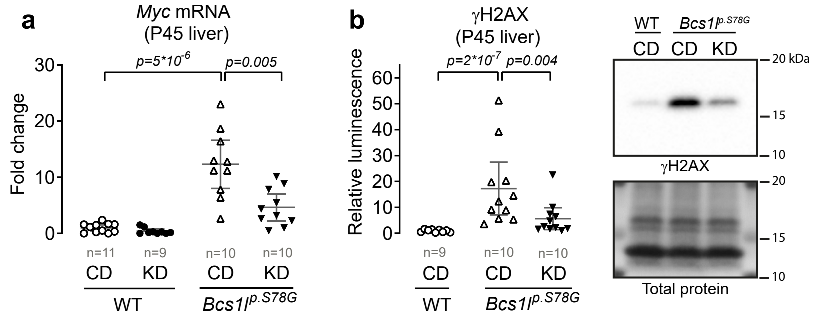


**Supplementary Fig. 8 | The effect of ketogenic diet (KD) on hepatic *Myc* expression and H2AX phosphorylation. a**, c-Myc mRNA (RNAseq). CD, control diet. **b**, Western blot quantification of γH2AX from P45 liver lysates and a representative blot. The quantified samples were run in a randomized order on two 26-well gels followed by parallel Western blots. Statistics: 1-way ANOVA followed by the selected pairwise comparisons (Welch's t-statistics). The error bars represent 95% CI of mean. All data points derive from independent mice. Source data are provided as a Source Data file.

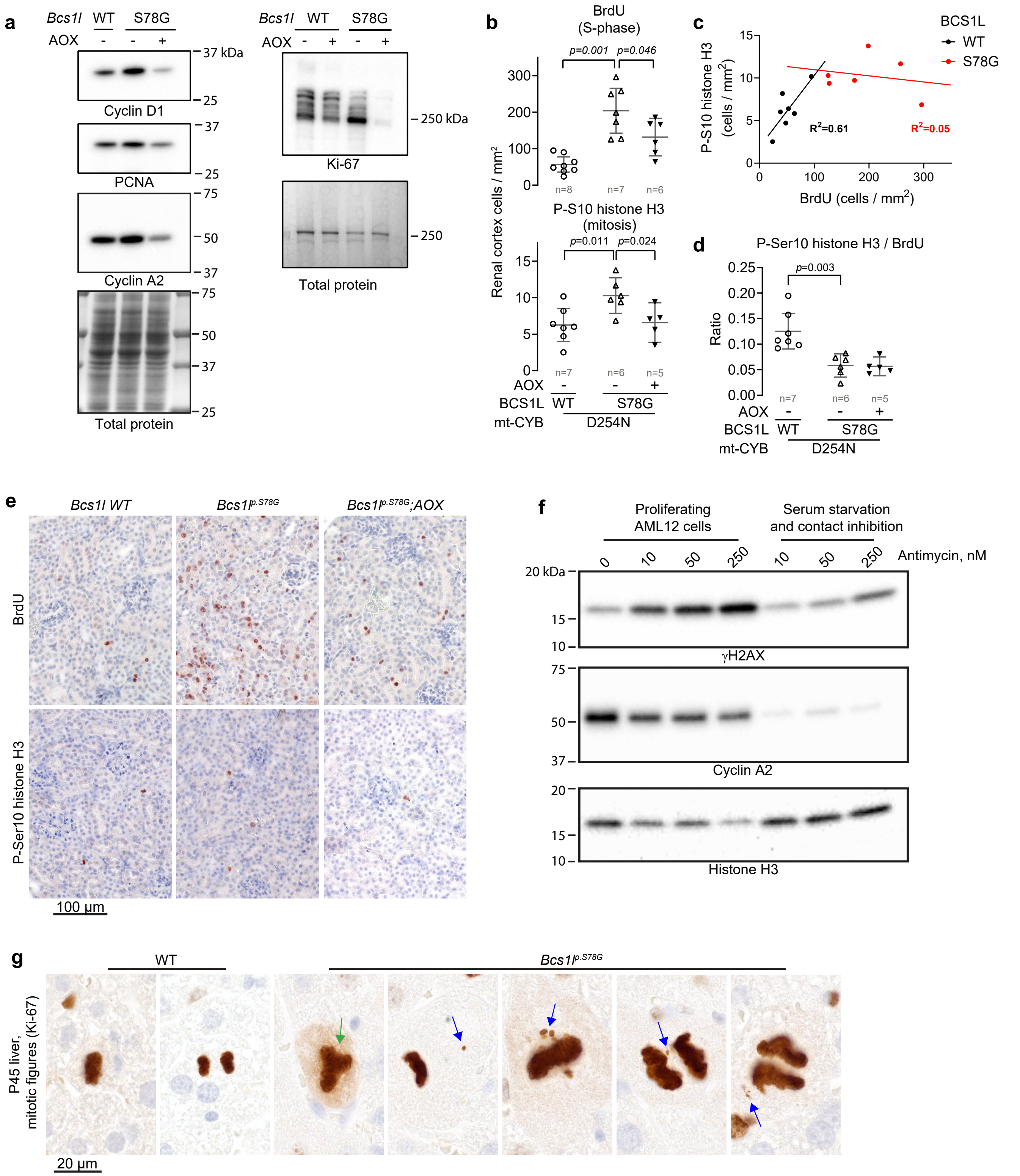


**Supplementary Fig. 9 | Assessment of cell cycle in *Bcs1l*$^{p.S78G}$*;mt-Cyb*$^{p.D254N}$ tissues and the effect of antimycin A on DNA damage in proliferating cultured cells. a** Representative Western blot detections of the indicated proliferating cell markers (quantifications shown in Fig. 9a) from P30 liver lysates. The data are from the mice of *mt-Cyb*$^{p.D254N}$ mtDNA background. **b** Quantification of replicating cells in P30 renal cortex based on BrdU incorporation (S-phase) and Histone H3 Ser-10 phosphorylation (mitosis). **c** Correlation between the number of BrdU- and histone H3 phosphorylation-positive renal cortex epithelial cells, showing a relative lack of mitotic cells in *Bcs1l*$^{p.S78G}$*;mt-Cyb*$^{p.D254N}$ mice. **d** Ratio of histone H3 P-Ser10- to BrdU-positive cells. **e** Representative images of renal cortex immunostained for BrdU or histone H3 P-Ser10. **f** Effect of the CIII inhibitor antimycin A on γH2AX levels in AML12 cells in growth phase and under suppression of proliferation by serum starvation and contact inhibition (representative result of more than three similar experiments). The suppression of cellular proliferation was verified by assessing cyclin A2 levels. Histone H3 served as a loading control. **g**, Aberrant mitotic figures in P45 liver sections of *Bcs1l*$^{p.S78G}$ mice immunostained for Ki-67. Green arrow, multipolar mitotic spindle; blue arrows, lagging or aberrantly dispersed chromosomes or chromatin fragments. Statistics: 1-way ANOVA followed by the selected pairwise comparisons. The error bars represent 95% CI of mean. All data points derive from independent mice. Source data are provided as a Source Data file.

**Supplementary Table 1. Antibodies**

| Target | Antibody type | Source | Identifier | Dilution in WB | Dilution in IHC |
|---|---|---|---|---|---|
| CDKN1A | Rabbit monoclonal | Abcam | Clone EPR18021, ab188224 | 0.125-0.25 µg/ml | |
| Cleaved histone H3 | Rabbit monoclonal | Cell Signaling Technology | Clone D7J2K, 12576 | 1:2000-1:4000 | |
| Histone H3 | Mouse monoclonal | Cell Signaling Technology | Clone 96C10, 3638 | 1:4000 | |
| GPNMB (IHC) | Goat polyclonal | Santa Cruz | sc-47006 | | 0.5 µg/ml |
| GPNMB (WB) | Rabbit monoclonal | Cell Signaling Technology | Clone E7U1Z, 90205 | 1:4000 | |
| γH2AX | Rabbit monoclonal | Abcam | Clone EP854(2)Y, ab81299 | 70 ng/ml | 70 ng/ml |
| 53BP1 | Rabbit polyclonal | Novus Biologicals | NB100-304 | | 0.2 µg/ml |
| Lamin A/C | Rabbit polyclonal | Cell Signaling Technology | 2032 | 1:2000-1:4000 | 1:1000 |
| Lamin A/C (Supplementary Fig. 2f) | Mouse monoclonal | Cell Signaling Technology | Clone 4C11, 4777 | 1:8000 | |
| TP53 (p53) | Mouse monoclonal | Cell Signaling Technology | Clone 1C12, 2524 | 1:2000-1:4000 | |
| UQCRFS1 | Mouse monoclonal | Abcam | Clone 5A5, ab14746 | 0.25 µg/ml | |
| UQCRC1 | Mouse monoclonal | Abcam | Clone 16D10AD9AH5, ab110252 | 0.25 µg/ml | |
| PRDX3 | Rabbit polyclonal | Abcam | ab73349 | 0.5 µg/ml | |
| PRDX1 | Rabbit monoclonal | Abcam | Clone EPR5433, ab109498 | 12 ng/ml | |
| DHODH | Rabbit polyclonal | Proteintech | 14877-1-AP | 42 ng/ml | |
| VDAC1 | Mouse monoclonal | Abcam | clone 20B12AF2, ab14734 | 0.2 µg/ml | |
| P(Ser51)-eIF2α | Rabbit monoclonal | Cell Signaling Technology | Clone 119A11, 3597 | 1:4000 | |
| eIF2α | Rabbit polyclonal | Cell Signaling Technology | 9722 | 1:4000 | |
| ATF4 | Rabbit monoclonal | Cell Signaling Technology | Clone D4B8, 11815 | 1:4000 | |
| P(S240/244)-RPS6 | Rabbit polyclonal | Cell Signaling Technology | 2215 | 1:4000 | |
| RPS6 | Rabbit monoclonal | Cell Signaling Technology | Clone 5G10, 2217 | 1:4000 | |
| c-MYC | Rabbit monoclonal | Abcam | Clone Y69, ab32072 | 0.1 µg/ml | |
| P(Thr172)-AMPKα | Rabbit monoclonal | Cell Signaling Technology | Clone 40H9, 2535 | 1:4000 | |
| AMPKα | Rabbit monoclonal | Cell Signaling Technology | Clone 23A3, 2603 | 1:2000 | |
| Cyclin D1 | Rabbit monoclonal | Cell Signaling Technology | Clone E3P5S, 55506 | 1:4000 | |
| PCNA | Mouse monoclonal | Dako | Clone PC10, M0879 | 1:2000-1:3000 | |
| Cyclin A2 | Rabbit monoclonal | Abcam | Clone EPR17351, ab181591 | 0.125 µg/ml | 0.5 µg/ml |
| Ki67 | Rabbit monoclonal | Abcam | Clone SP6, ab16667 | 15 ng/ml | |
| P(Ser10)-histone H3 | Rabbit polyclonal | Cell Signaling Technology | 9701 | | 1:1000 |
| GFP | Rabbit polyclonal | Invitrogen | A-11122 | 0.4 µg/ml | 0.67 µg/ml |
| Omomyc | Rabbit polyclonal | Prof. Laura Soucek's laboratory | | 1:5000 | 1:2000 |
| BrdU | Mouse monoclonal | Dako | Clone Bu20a, M0744 | | 1.1 µg/ml |
| Rabbit IgG | Goat polyclonal, peroxidase conjugate | Cell Signaling Technology | 7074 | 1:2000-1:15000 | |
| Mouse IgG | Goat polyclonal, peroxidase conjugate | Cell Signaling Technology | 7076 | 1:2000-1:15000 | |
| Mouse IgG light chain | Rabbit polyclonal, peroxidase conjugate | Cell Signaling Technology | 58802 | 1:4000 | |

WB, Western blot; IHC, immunohistochemistry;

**Supplementary Table 2. Oligonucleotide sequences in 5' to 3' direction**

| qPCR primers | Forward primer | Reverse primer |
|---|---|---|
| *Cdkna1* | CGAGAACGGTGGAACTTTGAC | CCAGGGCTCAGGTAGACCTT |
| *Ctsl* | CCCTATGAAGCGAAGGACGG | CTGGAGAGACGGATGGCTTG |
| *Gpnmb* | AAAATTGCTGACCACTTAGGACA | TCTCCAAACATTGCCAAACA |
| *Cdkn2a variant 1* (*p19$^{ARF}$*) | TGTTGAGGCTAGAGAGGATCTTG | CGAATCTGCACCGTAGTTGAGC |
| *Cdkn2a variant 2* (*p16$^{INK4a}$*) | TCACACGACTGGGCGATTG | TGCCCATCATCATCACCTGAATC |
| *Lamin C* | CGACGAGGATGGAGAAGAGC | AGACTTTGGCATGGAGGTGG |
| *Lamin A* (71 bp amplicon) | AGAGATGGGAATGACGGGGA | CAGGCTCAGAAGCTGGGAAA |
| *Lamin A* (primers flanking the progerin deletion) | CGACGAGGATGGAGAAGAGC | TGATGCTGCAGTTCTGGGAG |
| *Progerin* | CGACGAGGATGGAGAAGAGC | AGTTCTGGGAGCTCTGGGCT |
| *18s rRNA* | ACCCGTTGAACCCCATTCGTGA | GCCTCACTAAACCATCCAATCGG |
| *Psat1* | CTTAGCACCATGGAAGCCAC | TGACTCATTTCGAGCACACTGA |
| *Phgdh* | ATGTCATCAGCTGTCCCCAC | GCGTTCACAACGCCTGTTAG |
| *Tyms* | TTATGCTGGTGGTTGGCTCC | AGGAAATTCATCTCTCAGGCTGT |
| *Ctps* | AGCCGGCCTCAGGTTAAAA | GCTGGCGATGACTCCTTTTC |
| *Dpys* | GAAGATGACACTGCTGGAACC | TCTTGGCTGTCTTTCAGAAACC |
| *Dpyd* | CTGTGGGCATTGGAAGGA | CAATGGGCCTGATAGCAGTT |
| *Tymp* | GGCCCCTGAATTGAATAGACTGG | GATGCCCTCCATCTCGCTTC |
| *Atf4* variant 1* (NM_009716.3) | TGCCGGTTTAAGTTGTGTGC | GGATTTCGTGAAGAGCGCCAT |
| *Atf4* variant 2* (NM_001287180.1) | CCTATAAAGGCTTGCGGCCA | GATTTCGTGAAGAGCGCCAT |
| *Atf3* | GTCACCAAGTCTGAGGCGG | CGGTGTCCGTCCATTCTGAG |
| *Atf5* | ATGAAGAGGAATAAGATGAGGTCC | AGGCAGCGTGGAAGATTGTT |
| *Trib3* | AGCACTTTAGCAGCGGAAGA | AGGTGTAGCTCGCATCTTGT |
| *Ddit3* | CAGGAGAACGAGCGGAAAGT | GAGACAGACAGGAGGTGATGC |
| *Asns* | CGCTAGTGCTCAGAGTGCC | CACTCAGACACTGCACGGAA |
| *Mthfd2* | TTTCCTTGTTGTCTGCGTTGG | AACGGCTTCATTTCGCACT |
| *Gdf15* | GTCTCCCCGAAGCCTACC | TTCAGGGGCCTAGTGATGTC |
| *Myc* | GCGACTCTGAAGAAGAGCAAG | GCCTCGGGATGGAGATGAG |
| *Gak* | CTGCCCACCAGGCATTTG | CCATGTCACATACATATTCAATGTACCT |
| *Rab11a* | AAGGCACAGATATGGGACACA | CCTACTGCTCCACGATAGTATGC |
| Nuclear DNA (mtDNA copy number qPCR) | AAAGTGGGAATCTGGACACG | CAGAGGCCTTATTTCATTTTCG |
| mtDNA (mtDNA copy number qPCR) | TCCTACTGGTCCGATTCCAC | GGCTCCGAGGCAAAGTATAG |

| Other oligonucleotides | Sequence |
|---|---|
| dGTP detection template (dNTP assay) | GGAGTGAGTGTGAGGTGAATGGGGAGTGAGTGTGAGGTGAATGGGGAGTGAGT GTGAGGTGAATGGGGAGTGAGTGTGAGGTGAATGGGGAGTGAGTGTGAGGTGA ATGGGGAGTGAGTGTGAGGTGAATGGGGAGTGAGTGTGAGGTGAATGGGGAGT GAGTGTGAGGTGAATGGTTTCTTTGGCGGTGGAGGCGG |

* Both splice variants showed near-identical expression patterns. Thus, geometric mean of expression of the variants are shown in Fig. 7a
