# Supplementary material for: Mitochondrial complex III deficiency drives c-MYC overexpression and illicit cell cycle entry leading to senescence and segmental progeria
Source: Nat Commun. 2023 Apr 24;14:2356. doi: 10.1038/s41467-023-38027-1 (PMC10126100; doi:10.1038/s41467-023-38027-1)
Supplement: Supplementary file 3 — Reporting Summary [file 41467_2023_38027_MOESM3_ESM.pdf]

nature portfolio

Corresponding author(s): Jukka Kallijärvi

Last updated by author(s): Mar 31, 2023

Reporting Summary

Nature Portfolio wishes to improve the reproducibility of the work that we publish. This form provides structure for consistency and transparency in reporting. For further information on Nature Portfolio policies, see our Editorial Policies and the Editorial Policy Checklist.

Statistics

For all statistical analyses, confirm that the following items are present in the figure legend, table legend, main text, or Methods section.

n/a	Confirmed	
☐	☒	The exact sample size (n) for each experimental group/condition, given as a discrete number and unit of measurement
☐	☒	A statement on whether measurements were taken from distinct samples or whether the same sample was measured repeatedly
☐	☒	The statistical test(s) used AND whether they are one- or two-sided<br>*Only common tests should be described solely by name; describe more complex techniques in the Methods section.*
☐	☒	A description of all covariates tested
☐	☒	A description of any assumptions or corrections, such as tests of normality and adjustment for multiple comparisons
☐	☒	A full description of the statistical parameters including central tendency (e.g. means) or other basic estimates (e.g. regression coefficient) AND variation (e.g. standard deviation) or associated estimates of uncertainty (e.g. confidence intervals)
☐	☒	For null hypothesis testing, the test statistic (e.g. F, t, r) with confidence intervals, effect sizes, degrees of freedom and P value noted<br>*Give P values as exact values whenever suitable.*
☒	☐	For Bayesian analysis, information on the choice of priors and Markov chain Monte Carlo settings
☒	☐	For hierarchical and complex designs, identification of the appropriate level for tests and full reporting of outcomes
☐	☒	Estimates of effect sizes (e.g. Cohen's d, Pearson's r), indicating how they were calculated

Our web collection on statistics for biologists contains articles on many of the points above.

Software and code

Policy information about availability of computer code

Data collection	Zeiss Zen software version 2.3 (microscopy images), Bio-Rad Image Lab version 6.1 (Western blot acquisition), Bio-Rad CFX Manager version 3.1 (qPCR), Biotek Gen5 version 3.08 (data acquisition for microplate assays), Oroboros DatLab version 6 (Oxygraphy). The collection of RNAseq data is described in the original publications as referred in the Materials and Methods.
Data analysis	IBM SPSS Statistics versions 26-29 (statistics), GraphPad Prism versions 8.0-9.5 (data presentation and survival analysis), Bio-Rad CFX Manager version 3.1 (qPCR), Bio-Rad CFX Maestro version 2.2.1 (qPCR), Bio-Rad Image Lab versions 5.2.1 and 6.1 (Western blot), Fiji-ImageJ versions 1.52-1.53 (image analyses), Adobe Photoshop versions 20-23 (image analyses), Gitools version 2.3.1 (http://www.gitools.org) (heatmaps).

For manuscripts utilizing custom algorithms or software that are central to the research but not yet described in published literature, software must be made available to editors and reviewers. We strongly encourage code deposition in a community repository (e.g. GitHub). See the Nature Portfolio guidelines for submitting code & software for further information.

Data

Policy information about availability of data

All manuscripts must include a data availability statement. This statement should provide the following information, where applicable:

- Accession codes, unique identifiers, or web links for publicly available datasets
- A description of any restrictions on data availability
- For clinical datasets or third party data, please ensure that the statement adheres to our policy

The numerical data, and uncropped representative Western blots and the related blots used for quantifications are available as Source Data file accompanying this

paper. The following public datasets available from ArrayExpress database were utilized in this study: E-MTAB-741626 and E-MEXP-150340.

Human research participants

Policy information about studies involving human research participants and Sex and Gender in Research.

Reporting on sex and gender	n/a
Population characteristics	n/a
Recruitment	n/a
Ethics oversight	n/a

Note that full information on the approval of the study protocol must also be provided in the manuscript.

Field-specific reporting

Please select the one below that is the best fit for your research. If you are not sure, read the appropriate sections before making your selection.

[x] Life sciences   [ ] Behavioural & social sciences   [ ] Ecological, evolutionary & environmental sciences

For a reference copy of the document with all sections, see nature.com/documents/nr-reporting-summary-flat.pdf

Life sciences study design

All studies must disclose on these points even when the disclosure is negative.

Sample size	Predetermination of main response variable and its sample size was not meaningful due to the nature of this study. For the AOX intervention, a priori power analysis (80% power, 0.05 significance) indicated that to be able to detect 20% increase in the survival of Bcs1l mutant mice, we would need at least 5 mice per group, both genders included. For most of the biochemical assays we had no a priori knowledge on variation, and therefore it was not possible to perform power calculations to estimate sample sizes. The sample sizes in many biochemical analyses (e.g. Western blot analyses) were partly determined by the nature of the method. We aimed for maximal feasible and available sample sizes for the given method, e.g. 25 protein samples in one large SDS-PAGE gel, typically 7-9 samples per group/genotype. We were interested only in robust changes, and we base our conclusions on highly significant robust effects sizes as can be seen from the figures. Moreover, not a single statistical comparison is critical for the main conclusions of this study. Thus, the sample sizes were sufficient.
Data exclusions	In Fig. 4j, two extreme outliers (+38.8 SD and +8.5 SD differences to the mean value of mice of the same genotype) were excluded due to the lack of similar variation in replication experiments (Source Data file). In Fig. 6c, one extreme outlier (+13.6 SD difference to the mean value of mice of the same genotype) was excluded from descriptive statistics, but this data point is shown in the figure itself as described in the figure legends. Regarding Fig. 8a, one extreme outlier (+28.7 SD difference to the mean value of mice of the same genotype) with a strong suspicion of incorrect genotyping result was excluded. Regarding Fig. 10 b,c, two rAAV-injected mice were excluded prior to the data analysis due to the lack of detectable expression of the recombinant protein. The excluded data points are included in the Source Data.
Replication	We analyzed several tissues from two different mouse strains and mice of different ages to corroborate the robustness of the findings. We utilized maximal feasible sample sizes for most assays. We utilized several different approaches to examine the biological processes of interest (mouse phenotyping, histology; and mRNA, protein and metabolite level data) so that no single parameter is critical for our main conclusions. Where the number of biological replicates were minimal e.g. cell culture work, the experiments were repeated at least three times. All technically successful replication experiments showed consistent results.
Randomization	The Bcs1l genotypes were randomized by the Mendelian rules. The experiment was designed so that other genetic factors were essentially fully controlled. The order of sample collection and fresh sample analyzes was dictate by the birth date of the mice. All stored samples were analyzed in randomized order.
Blinding	The personnel evaluating the mice were blinded to the genotype information. However, due to striking size difference, Bcs1l mutant mice can be easily distinguished from wild-type littermates. The data were collected and analyzed without awareness to group allocation, though, no strict blinding was applied.

Reporting for specific materials, systems and methods

We require information from authors about some types of materials, experimental systems and methods used in many studies. Here, indicate whether each material, system or method listed is relevant to your study. If you are not sure if a list item applies to your research, read the appropriate section before selecting a response.

Materials & experimental systems

n/a	Involved in the study
☐	☒ Antibodies
☐	☒ Eukaryotic cell lines
☒	☐ Palaeontology and archaeology
☐	☒ Animals and other organisms
☒	☐ Clinical data
☒	☐ Dual use research of concern

Methods

n/a	Involved in the study
☒	☐ ChIP-seq
☒	☐ Flow cytometry
☒	☐ MRI-based neuroimaging

Antibodies

Antibodies used

Supplementary Table 1 list the antibodies used and their identifiers.

Antibodies from Abcam:
CDKN1A (Clone EPR18021, ab188224) (0.125-0.25 µg/ml, WB)
yH2AX (Clone EP854(2)Y, ab81299) (70 ng/ml, WB and IHC)
UQCRFS1 (Clone 5A5, ab14746) (0.25 µg/ml WB)
UQCRC1 (Clone 16D10AD9AH5, ab110252) (0.25 µg/ml, WB)
PRDX3 (ab73349) (0.5 µg/ml, WB)
PRDX1 (Clone EPR5433, ab109498), (12 ng/ml, WB)
VDAC1 (clone 20B12AF2, ab14734) (0.2 µg/ml, WB)
c-MYC (Clone Y69, ab32072), (0.1 µg/ml, WB)
Cyclin A2 (Clone EPR17351, ab181591) (0.125 µg/ml WB; 0.5 µg/ml, IHC)
Ki67 (Clone SP6, ab16667) (15 ng/ml, WB)

Antibodies from Cell Signaling Technologies
Cleaved histone H3 (Clone D7J2K, 12576) (1:2000-1:4000, WB)
Histone H3 (Clone 96C10, 3638), (1:4000, WB)
GPNMB (Clone E7U1Z, 90205) (1:4000 WB)
Lamin A/C (2032) (1:2000-1:4000, WB; 1:1000 IHC)
Lamin A/C (Clone 4C11, 4777), (1:8000, WB)
TP53 (Clone 1C12, 2524) (1:2000-1:4000, WB)
P(Ser51)-eIF2-alpha (Clone 119A11, 3597) (1:4000, WB)
eIF2-alpha (9722) (1:4000 WB)
ATF4 (Clone D4B8, 11815) (1:4000, WB)
P(S240/244)-RPS6 (2215) (1:4000, WB)
RPS6 (Clone 5G10, 2217) (1:4000 WB)
P(Thr172)-AMPK-alpha (Clone 40H9, 2535) (1:4000, WB)
AMPK-alpha (Clone 23A3, 2603) (1:2000, WB)
Cyclin D1 (Clone E3P5S, 55506) (1:4000, WB)
P(Ser10)-histone H3 (9701) (1:1000, IHC)
Anti-rabbit IgG (7074) (1:2000-1:15000, WB)
Anti-mouse IgG (7076) (1:2000-1:15000, WB)
Anti-mouse IgG light chain (58802) (1:4000, WB)

Antibodies from Dako
PCNA (Clone PC10, M0879) (1:2000-1:3000, WB)
BrdU (Clone Bu20a, M0744) (0.1 µg/ml, dot blot; 1.1 µg/ml IHC)

Antibodies from other sources
GFP (Invitrogen, A-11122) (0.4 µg/ml, WB; 0.67 µg/ml IHC)
53BP1 (Novus Biologicals,NB100-304) (0.2 µg/ml, IHC)
Omomyc (Custom antibody produced by Prof. Laura Soucek's laboratory) (1:5000, WB; 1:2000, IHC)
DHODH (Proteintech,14877-1-AP) (42 ng/ml, WB)
GPNMB (Santa Cruz,sc-47006) (0.5 µg/ml, IHC)

Validation

High-quality antibodies from reputable manufacturers and with extensive validation data (knock-out-validated whenever available) and citations were used. The antibodies were further validated technically in-lab and accepted only if they gave a single band or band pattern of predicted size in Western blot, as shown by the manufacturers validation data and relevant publications, and/or if possible non-specific bands were clearly possible to discriminate from the specific one(s). mRNA expression changes between genotypes observed in transcriptomics data or by qPCR were one validation criterium. GPNBM antibody was validated by reproducing similar staining of the foci with another antibody. BrdU immunodetections were validated by parallel staining of control samples from

unexposed mice. Omomyc and GFP antibodies were validated by parallel staining of control samples lacking expression of these proteins. In immunohistochemical staining, irrelevant control antibody of same immunoglobulin class was always used.

Eukaryotic cell lines

Policy information about cell lines and Sex and Gender in Research

Cell line source(s)	AML12 mouse hepatocyte line, source ATCC cat no. CRL-2254
Authentication	Not authenticated because of the immediate commercial source.
Mycoplasma contamination	The cell line was not tested for mycoplasma after it was purchased from ATCC. No mycoplasma contaminations were detected in the cell culture facility during the experiments.
Commonly misidentified lines (See ICLAC register)	n/a

Animals and other research organisms

Policy information about studies involving animals; ARRIVE guidelines recommended for reporting animal research, and Sex and Gender in Research

Laboratory animals	Mouse (Mus musculus) C57BL/6JCrl (Harlan stock 000664), age between P19-P220. All mice received water and chow (Teklad 2018, Harlan) ad libitum. The animal facilities of University of Helsinki housed the mice in temperature-controlled (23oC) individually-ventilated cages under a 12-h light/dark cycle. The relative humidity in the facility was maintained between 45-65%.
Wild animals	n.a.
Reporting on sex	The analyses included both sexes unless otherwise stated. Where appropriate, the figures show the data individually for both sexes.
Field-collected samples	n.a.
Ethics oversight	The animal ethics committee of the State Provincial Office of Southern Finland approved the animal studies (permit numbers ESAVI/6365/04.10.07/2017 and ESAVI/16278/2020). We performed the animal experiments according to the FELASA (Federation of Laboratory Animal Science Associations) guidelines and best practices.

Note that full information on the approval of the study protocol must also be provided in the manuscript.
